# Radiographic Assessment of Aseptic Loosening of Tumor‐Type Knee Prosthesis in Distal Femur

**DOI:** 10.1111/os.13297

**Published:** 2022-05-07

**Authors:** Zi‐ming Li, Xiu‐chun Yu, Kai Zheng

**Affiliations:** ^1^ First Clinical Medical College Shandong University of Traditional Chinese Medicine Jinan China; ^2^ Department of Orthopaedics The 960th Hospital of the PLA Jinan China

**Keywords:** Alignment, Aseptic loosening, Distal femur, Endoprosthetic replacement, Stem

## Abstract

**Objective:**

To measure the full‐length anteroposterior and lateral radiographs of lower limbs after the resection of a tumor in the distal femur and tumor‐type knee prosthesis replacement and to analyze the factors leading to aseptic loosening of the prosthesis.

**Methods:**

A total of 26 cases of tumor‐type knee prosthesis replacement or revision due to the distal femoral tumor at our hospital from January 2007 to December 2019 were retrospectively analyzed. The patients were divided into the loosening and unloosening groups depending on whether aseptic loosening occurred after surgery. Full‐length anteroposterior and lateral radiographs of lower limbs were used to measure bone resection length, length of prosthesis, distance of proximal apex of the medullary stem of the femoral prosthesis from the maximum arc of the anterior femoral arch, diameter of the medullary stem, etc. Data were analyzed, and the risk factors for aseptic loosening of the prosthesis were explored.

**Results:**

The ratio of the prosthetic length to the femoral length (63.72 ± 5.21) and the ratio of the femoral medullary stem diameter to the femoral diameter (26.03 ± 8.45) were smaller in the loosening group than in the unloosening group. The difference was statistically significant (*p* < 0.05). The distance between the apex of the medullary stem and the maximum arc of the anterior femoral arch was significantly shorter in the loosening group (3.47 ± 2.96) than in the unloosening group, and the difference was statistically significant (*p* < 0.05). The measurement of the lower limb alignment showed significant differences between the loosening and unloosening groups in terms of HKAA, mLDFA, and distance between the lower limb alignment and the center of the knee joint (*p* < 0.05). The logistic regression analysis showed that less than 30% ratio between the medullary stem diameter and the femoral diameter, less than 3 cm distance between the apex of the medullary stem and the maximum curvature of the anterior arch of the femur, distance between the lower limb alignment and the center of the knee joint, and presence of varus knee and valgus knee after the surgery were the risk factors for aseptic loosening of the prosthesis.

**Conclusions:**

The diameter of the femoral medullary stem of the prosthesis, the apex position of the prosthetic stem, and the lower limb alignment are the risk factors for aseptic loosening of the prosthesis.

## Graphical abstract

The boxplot indicates that the distance between the apex of medullary stalk and the maximum arc of anterior femoral arch in the loosening group was shorter than that in the unloosening group and the difference was statistically significant.

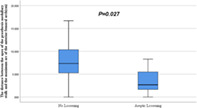

## Introduction

Malignant bone tumors occurring around the knee joint can seriously affect quality of life. Limb‐sparing surgery, represented by the resection of tumor and prosthesis replacement, has become the surgical modality for treating malignant bone tumors occurring around the knee joint^1^, ^2^, ^3^. The advantages of tumor prosthesis include good early‐stage reliability, rapid postoperative recovery, better cosmetic effect, improved psychological acceptance by patients, and satisfactory limb function. However, significant individual differences exist in the survival time of prostheses in clinical practice^4^, ^5^. Other studies have confirmed aseptic loosening as the primary cause of the long‐term failure of distal femoral tumor prosthesis failure. Some patients require second revision surgery due to aseptic loosening of the prosthesis^6^, ^7^. The causes of aseptic loosening of tumor‐type prostheses and reduction of the incidence of aseptic loosening are urgent clinical issues that need to be resolved.

Different views exist on the specific causes of aseptic loosening of tumor‐type prostheses. Song *et al*.^8^ suggested that the length of bone resection was related to prosthesis loosening and bone resection negatively correlated with implant longevity. Slone *et al*.^9^ proposed that concentrated stresses in the intramedullary stem and the prosthetic binding site caused loosening. Lu *et al*.^10^ demonstrated that the mismatch between the straight medullary cavity stem and the femoral anterior curvature also led to the failure of the prosthesis. Reducing the incidence of aseptic loosening was one of the concerns.

Various methods have been proposed to avoid aseptic loosening in tumor‐type knee prosthesis replacement surgery. Zhang *et al*.^11^ observed a nonlinear relationship and an optimal inflection point between the length of the femoral stem and the risk of aseptic loosening, and proposed to reduce the prosthesis failure rate by controlling the length of the medullary stem at around 143 mm. Piakong *et al*.^12^ suggested that a curved stem with a larger diameter could reduce the revision rate. Bischel *et al*.^13^. proposed that a hexagonal medullary stem apex design might reduce the prosthesis failure rate. Stevenson *et al*.^14^ suggested that the addition of cortical plates at the junction between the femur and the prosthesis could effectively reduce the loosening rate. Parra *et al*.^15^ suggested that non‐cemented stems could reduce the prosthesis failure rate by reducing the stress at the bone–prosthesis interface. Relatively few objective indicators exist for the imaging evaluation of the risk factors for prosthetic loosening. Piakong *et al*.^12^ and Turcotte *et al*.^16^ attempted to find imaging evidence of prosthetic loosening by studying the changes in the bone cement; however, measurements and studies on the prosthesis itself do not exist.

In the clinic, the length, diameter, and the apex position of the medullary stem were found to affect the occurrence of aseptic loosening of the prosthesis. The stem of prosthesis in the revision surgery was often shorter and thinner. The prosthetic stem was a key component connecting the prosthesis with the femur. It adhered to the femoral bone marrow cavity through a rough surface and cured bone cement. In theory, a longer prosthetic stem can obtain a larger contact area, which can reduce local stress and micromotion in the microenvironment and delay the occurrence of aseptic loosening. However, a longer medullary stem is often required during the surgery due to the location of the tumor and the principle of extensive osteotomy. The longer medullary stem must pass through the anterior femoral arch. The anterior femoral arch is the anterior curvature of the upper segment of the femur. The upper part of the femoral bone marrow cavity is the dorsal side, and the lower part of the medullary cavity is the anterior ventral side. In other words, the axis of the medullary cavity of the prosthesis produces different degrees of stress at the anterior femoral arch. The normal anterior femoral arch angle meets the requirements of the human weight line. The horizontal and vertical deviations of the knee prosthesis and the mutual adaptation between the femur and the medullary stem change with a change in the load conduction of the anterior arch angle of the femur, resulting in the disturbance of the lower extremity load conduction system. Studying the abnormal changes in the normal knee joint relationship caused by surgery has great clinical significance.

This study aimed to (i) identify the risk factors for aseptic loosening of the prosthesis by measuring the postoperative imaging data, including the proportion of bone resection, distance of the proximal apex of the femoral medullary stem from the maximum arc of the anterior femoral arch, ratio of the femoral medullary stem diameter to the femoral diameter, hip–knee–ankle angle (HKAA), mechanical lateral distal femoral angle (mLDFA), and mechanical medial proximal tibial angle (mMPTA); (ii) to verify the impact of the apex position of the prosthetic stem on the incidence of aseptic loosening; and (iii) to make recommendations for the reduction of the loosening rate.

## Patients and Methods

### Inclusion and Exclusion Criteria

The inclusion criteria were as follows: (i) patients who underwent rotary hinge knee prosthesis replacement for distal femoral bone tumor and those who underwent revision surgery for aseptic loosening of the prosthesis; (ii) bone‐cemented fixed prosthesis; (iii) postoperative full‐length X‐ray radiographs of the lower extremity; and (iv) available case data, including surgical method and pathology results.

The exclusion criteria were as follows: (i) composite tumor prosthesis, total femoral prosthesis, and extendable prosthesis; (ii) bone cement and biohybrid fixation; (iii) nonstandard ortholateral film information that could not be effectively measured; and (iv) patients lost to follow‐up and those with follow‐up time shorter than 12 months.

### Demographic Data

A total of 26 patients who underwent tumor‐type knee prosthesis replacement and revision surgery for distal femoral bone tumors in the Department of Orthopaedics of PLA 960th Hospital from January 2007 to December 2019 were included in the study.

Of the 26 patients, six developed aseptic loosening of the prosthesis after prosthetic replacement and underwent prosthetic revision surgery. They were assigned to the aseptic loosening group, where the shortest time since the revision surgery was 9 months and the longest time since the initial surgery was 18 years. These six patients were followed up for another 2–6 years after revision. Among them, five patients had good clinical outcomes without loosening and one patient had prosthesis loosening again 7 years after revision and underwent total femoral prosthesis replacement. At the end of the follow‐up of this study, the remaining 20 patients did not show symptoms and imaging of aseptic loosening. Therefore, they were included in the unloosened group. All 26 patients survived at the end of the follow‐up in this study.

The prostheses were all rotary hinge knee prostheses, 13 of which were customized prostheses (Beijing LDK Technology Company Ltd.) and 13 were assembled prostheses (Shandong WEGO Orthopedic Device Company Ltd.). They were made of either the titanium alloy or the cobalt–chromium–molybdenum alloy. The stems of the medullary cavity were designed with straight stems and apex cones. The fixation methods involved bone cement.

### Surgical Methods

The primary resection of the tumor in the distal femur and tumor‐type combined knee prosthesis replacement was considered as an example. After successful anesthesia, the patient was placed in the supine position. A medial parapatellar arthrotomy was performed, and soft tissues were elevated. The capsular tissues were released. The tumor was completely resected after cutting the proximal femur. A cavity was created in the cancellous bone of the proximal femur. Once the guide was secure, the arthritic articulating surface of the tibia was resected using an oscillating saw, the section of the tibia was removed, and the tibial component was positioned. The femoral intramedullary needle, femoral condyles component, and extension rod were positioned. After irrigation, the bony surfaces were dried, and polymethyl methacrylate bone cement was applied to the end of the femur and tibia. The actual femoral and tibial implants were then positioned and impacted for a perfect fit. A patch was used to cover the femoral implant. After motion assessment, a drainage tube was used, the subcutaneous tissue was closed, and the skin was approximated.

All surgeries were performed by the same surgeon and surgical team in our hospital. Therefore, the consistency of surgical methods was guaranteed to the greatest extent.

### Methods of Measurement

Postoperative full‐length X‐rays (weight‐bearing position) of both lower limbs of all patients were taken according to the measurement protocol of Ramadier *et al*.^17^. All patients stood on an X‐ray large‐plate multifunctional digital fluoroscopy system (Shimadzu‐Hama Narayaki II, Japan) with the lower leg pressed against the plate, knees straight, feet together and flat on the weight‐bearing plate, and both knees moderately internally rotated by about 10°–15° so that the small head of the fibula overlapped the tibia by about one‐third and the patella was oriented anteriorly. When the lateral film was taken, the patient was made to lie on the affected side, and the affected limb was tightly pressed against the plate and moderately rotated so that the posterior joint surface of the femoral condyles completed the overlap.

**Fig. 1:**
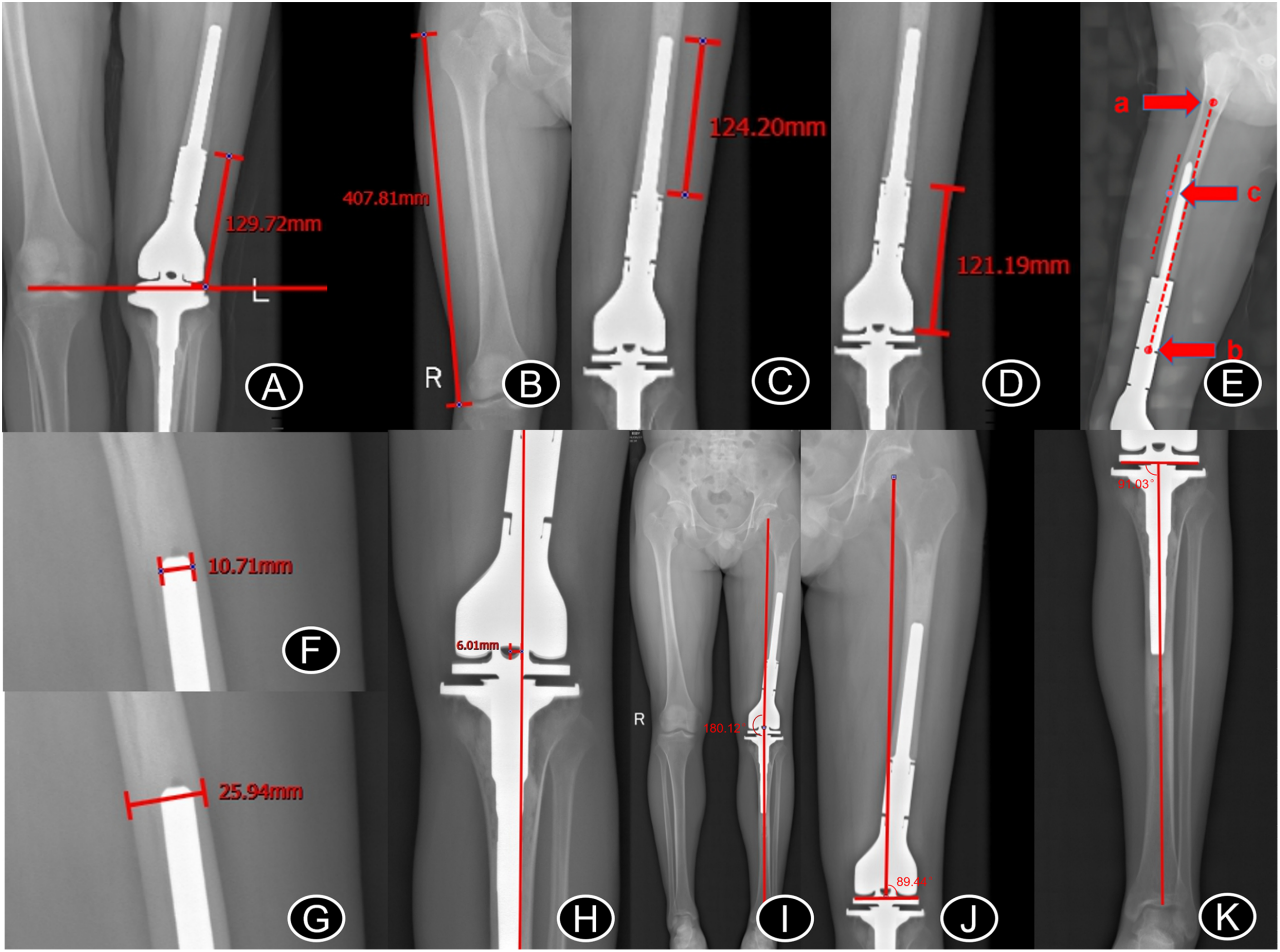
(A) Osteotomy length. (B) Femoral length. (C) Measurement of the length of the intramedullary part of the prosthesis. (D) Measurement of the extramedullary length of the prosthesis. (E) Selection of the point where the arc of the anterior femoral arch was maximum. Point a is the midpoint of the anterior and posterior cortex of the femur at the lower edge of the lesser trochanter, and point b is the midpoint of the anterior and posterior cortex at the level of 10 cm above the knee joint line. Point c is the point where the parallel line of a and b is tangent to the outermost cortex of the femur. (F) End diameter of the prosthesis medullary stem. (G) Femoral diameter at the end of the prosthesis medullary stem. (H) Distance between the lower limb force line and center of the knee joint. (I) HKAA. (J) mLDFA. (K) mMPTA. HKAA, hip–knee–ankle angle; mLDFA, mechanical lateral distal femoral angle; mMPTA, mechanical medial proximal tibial angle

The Picture Archiving and Communication System (AnyPacs v2.0; Medcare, Qingdao, China) with its own length and angle tools was used to measure the following data on the images:

- Osteotomy length, which is the distance from osteotomy to the knee joint level.
- Femoral length, which is the distance from the apex of the greater trochanter to the lateral femoral condyle.
- Length of the extramedullary portion of the prosthesis.
- Length of the intramedullary portion of the prosthesis.
- Distance of the proximal apex of the medullary stem of the femoral prosthesis from the maximum arc of the anterior femoral arch. In a standard lateral femoral radiograph, the line was drawn between point a (the midpoint of the anterior and posterior cortex of the femur at the lower edge of the lesser trochanter) and point b (the midpoint of the anterior and posterior cortex at a level of 10 cm above the knee joint line). Point c, where the parallel line was tangent to the most lateral cortices of the femur, was defined as the point where the arc of the anterior femoral arch was maximum (Figure 1E).
- Diameter of the femoral medullary stem of the prosthesis.
- Femoral diameter at the level of the end of the medullary stem of the prosthesis.
- Distance between lower limb alignment and center of the knee joint. The line between the center of the hip joint and the center of the ankle joint through the center of the knee joint was the lower limb alignment. Therefore, as long as the line deviated from the center of the knee joint, it represented an abnormal line of effort; the most common was varus or valgus deformity. A greater distance bias represented more serious varus or valgus deformity.
- HKAA, which was defined as the angle between the femoral mechanical axis and the tibial mechanical axis; a positive value represented valgus alignment, and the normal angle was 180° ± 3°.
- mLDFA, which is defined as the lateral angle between the femoral mechanical axis and the tangent to the femoral condyle; the normal angle was 87° ± 3°.
- mMPTA, which is defined as the medial angle between the tibial mechanical axis and the joint line of the proximal tibia; the normal angle was 87° ± 3°.

The aforementioned measurements were made, and the data were averaged. A method to locate the maximum arc of the anterior femoral arch was proposed based on the measurement methods of the anterior femoral arch angle and lateral arch angle. The method is shown in Figure 1E. The localization points selected by this method were compared with the localization points visually selected by three deputy chief physicians in our department. Among the 26 groups of localization points, 24 overlapped, indicating the clinical application of this method.

### Statistical Analysis

Statistical analysis was performed using SPSS 20.0 (SPSS Inc., Chicago, Illinois, USA). Normally distributed measures were expressed as mean ± standard deviation and analyzed using the *t* test for two independent samples. Non‐normally distributed measures were expressed as median (interquartile spacing) and analyzed using the Wilcoxon rank‐sum test to compare two independent samples. Binary logistic regression analysis was performed for risk factors, and odds ratio values and 95% confidence intervals were calculated.

## Results

### Comparison of General Data

The study included 16 men and 10 women, with age at presentation ranging from 15 to 66 years (mean, 33 years). The men‐to‐women ratio was 1.6:1. Among the patients, 16 had osteosarcoma, five had giant cell tumor of bone, one had chondrosarcoma, one had malignant fibroblastoma, one had malignant mesenchymal origin tumor, one had a primitive neuroectodermal tumor, and one had epithelioid hemangiosarcoma. The follow‐up time ranged from 2 to 18 years, with a median follow‐up time of 4.29 years.

The body mass index was higher in the loosening group (median, 25.80; range, 23.7–28.3) than in the unloosening group (median, 21.65; range, 17.9–34.6) with a significant difference (*p* < 0.05); however, significant differences were found in age, duration of surgery, intraoperative blood loss, follow‐up time, and other aspects (Table 1).

**TABLE 1:** Comparison of general data between aseptic loosening and no‐loosening groups (M [IQR])*

	Aseptic loosening	No loosening	*Z*	*p*
Age	38.5 (27)	26.5 (30)	−1.188	0.235
BMI	25.80 (2.4)	21.65 (4.4)	−2.861	0.004
Duration of surgery (min)	122.50 (69)	130.00 (41)	−0.092	0.927
Intraoperative blood loss (mL)	900.00 (875)	600.00 (500)	−1.382	0.16
Follow‐up^†^ (days)	3982.50 (5536)	1490.50 (1816)	−1.278	0.201

* M (IQR) is the median (interquartile spacing).

^†^ Follow‐up time in the loosening group referred to the interval between the initial replacement and the first revision surgery (day). Follow‐up time in the non‐loosening group referred to the interval between the initial replacement and December 31, 2020 (day)

Abbreviations: BMI, body mass index.

### Comparison of X‐Ray Measurements

**Fig. 2:**
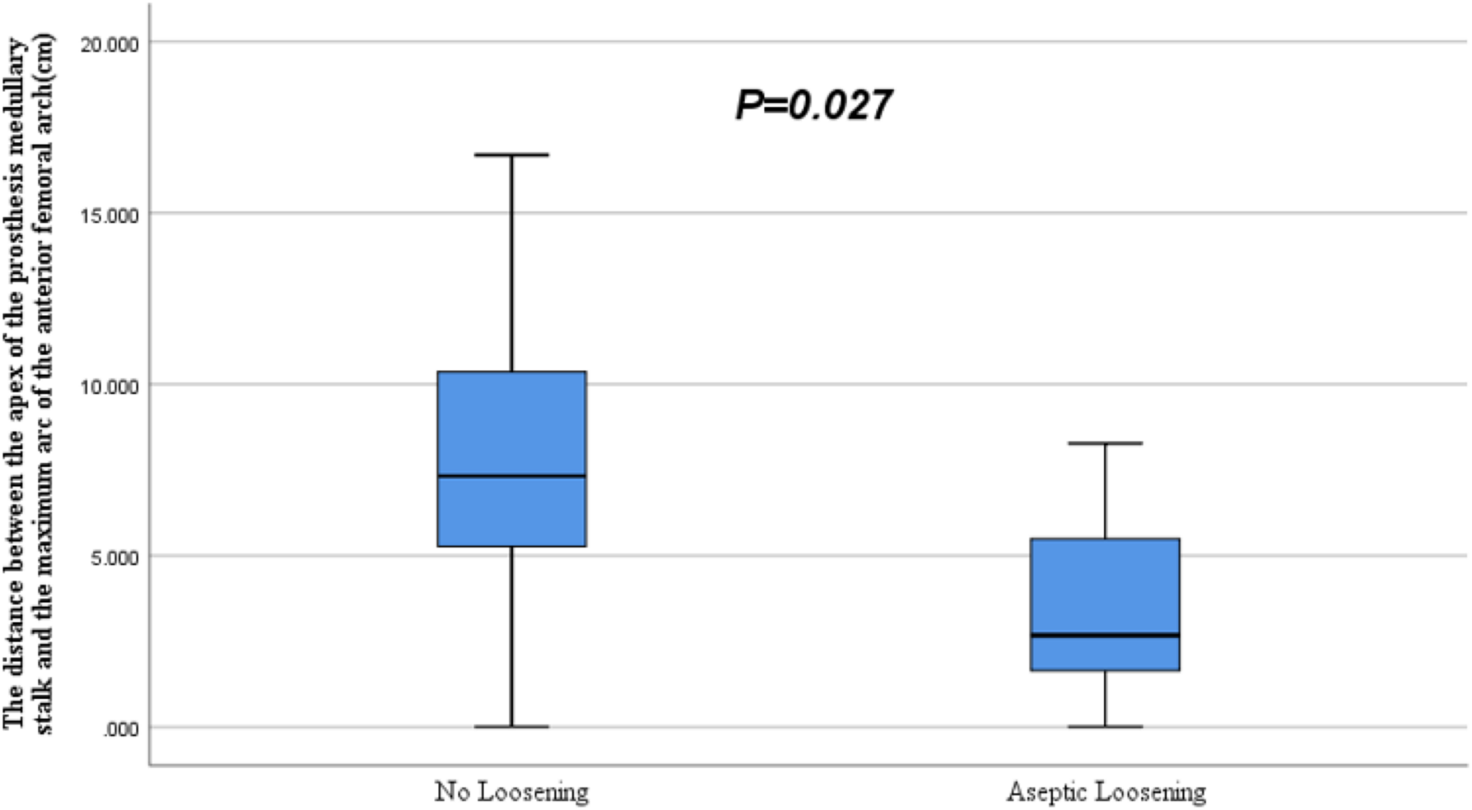
Distance between the apex of the medullary stem and the maximum curvature of the anterior femoral arch was statistically different between the two groups (*p* = 0.027)

Statistical analysis of the measured data from full‐length X‐ray films of lower limbs in the two groups showed that the ratio of the prosthetic length to the femoral length and the ratio of the femoral medullary stem diameter to the femoral diameter were smaller in the loosening group (63.72 ± 5.21; 26.03 ± 8.45) than in the unloosening group (73.92 ± 11.59; 36.83 ± 5.69). The differences were statistically significant (*p* < 0.05). Meanwhile, the distance between the apex of the medullary stem and the maximum arc of the anterior femoral arch was shorter in the loosening group (3.47 ± 2.96) than in the unloosening group (7.77 ± 3.40). The difference was statistically significant (*p* < 0.05) (Figure 2). No statistical difference was found in the ratio of osteotomy and the ratio of extramedullary to the intramedullary length of the prosthesis between the two groups (Table 2).

**TABLE 2:** Comparison of X‐ray measurement results of the aseptic loosening and no‐loosening groups

	Aseptic loosening	No loosening	*T*/*Z*	*p*
Proportion of bone resection (%，M [IQR])	33.20 (6.32)	37.25 (11.86)	−0.974	0.330
Ratio of the extramedullary length to the intramedullary length of the femoral prosthesis (M [IQR])	1.046 (0.26)	1.09 (0.44)	−0.791	0.429
Percentage of the prosthetic length to the femoral length (%，[mean ± SD])	63.72 ± 5.21	73.92 ± 11.59	−2.071	0.049
Percentage of the medullary stem diameter to the femoral diameter (%，[mean ± SD])	26.03 ± 8.45	36.83 ± 5.69	−2.936	0.024
Distance between the apex of the prosthesis medullary stem and the maximum arc of the anterior femoral arch (cm，[mean ± SD])*	3.47 ± 2.96	7.77 ± 3.40	−2.389	0.027

* In the unloosening group, the apex of the medullary stem did not exceed the maximum radian of the anterior femoral arch in four patients; therefore, the deleted value was not included in the statistics

### Lower Limb Alignment

The loosening group was statistically different from the unloosening group in terms of HKAA, mLDFA, and distance between the lower limb alignment and the center of the knee joint (*p* < 0.01, *p* < 0.05, and *p* < 0.01, respectively). No statistical difference existed in the mMPTA (Table 3).

**TABLE 3:** Comparison of lower limb alignment in the aseptic loosening and no‐loosening groups

	Aseptic loosening	No loosening	*t*/*Z*	*p*
HKAA (°，M [IQR])	175.83 (3.67)	180.189 (2.7)	−2.617	0.009
mLDFA (°，[mean ± SD])	87.50 ± 1.50	89.57 ± 1.72	−2.648	0.014
mMPTA (°，[mean ± SD])	89.08 ± 1.27	89.83 ± 2.18	−1.045	0.313
Distance between lower limb alignment and the center of the knee joint (cm，M [IQR])	1.54 (1.52)	0.02 (0.89)	−2.672	0.008

Abbreviations: HKAA, hip–knee–ankle angle; mLDFA, mechanical lateral distal femoral angle; mMPTA, mechanical medial proximal tibial angle.

### Risk Factors for Aseptic Loosening

Univariate factors with statistical differences were subjected to binary logistic regression analysis. The ratio of the medullary stem diameter to the femoral diameter of less than 30% (*p* < 0.05), the distance between the apex of the medullary stem and the maximum arc of the anterior femoral arch of less than 3 cm (*p* < 0.05), the distance between the lower extremity force line and the center of the knee joint (*p* < 0.05), and the presence of postoperative knee valgus and knee varus (*p* < 0.05) were the risk factors for the aseptic loosening of the prosthesis (Table 4).

**TABLE 4:** Risk factors affecting the aseptic loosening of the prosthesis: logistics analysis

Risk factor	OR	95% CI	*p*
BMI	1.376	0.974–1.861	0.072
Ratio of the prosthetic length to the femoral length	0.892	0.788–1.010	0.07
Ratio of the stem diameter to the femoral diameter was less than 30%	19.000	1.454–248.237	0.025
Relative distance between the apex of the medullary stem and the maximum curvature of the anterior femoral arch was less than 3 cm	11.333	1.395–92.056	0.023
Distance between lower limb alignment and the center of the knee joint	6.239	1.262–30.838	0.025
Abnormality in limb alignment*	11.333	1.395–92.056	0.023

* Varus knee (HKAA <178°) and valgus knee (HKAA >182).

Abbreviations: BMI, body mass index; HKAA, hip–knee–ankle angle; OR, odds ratio.

## Typical Case

**Fig. 3:**
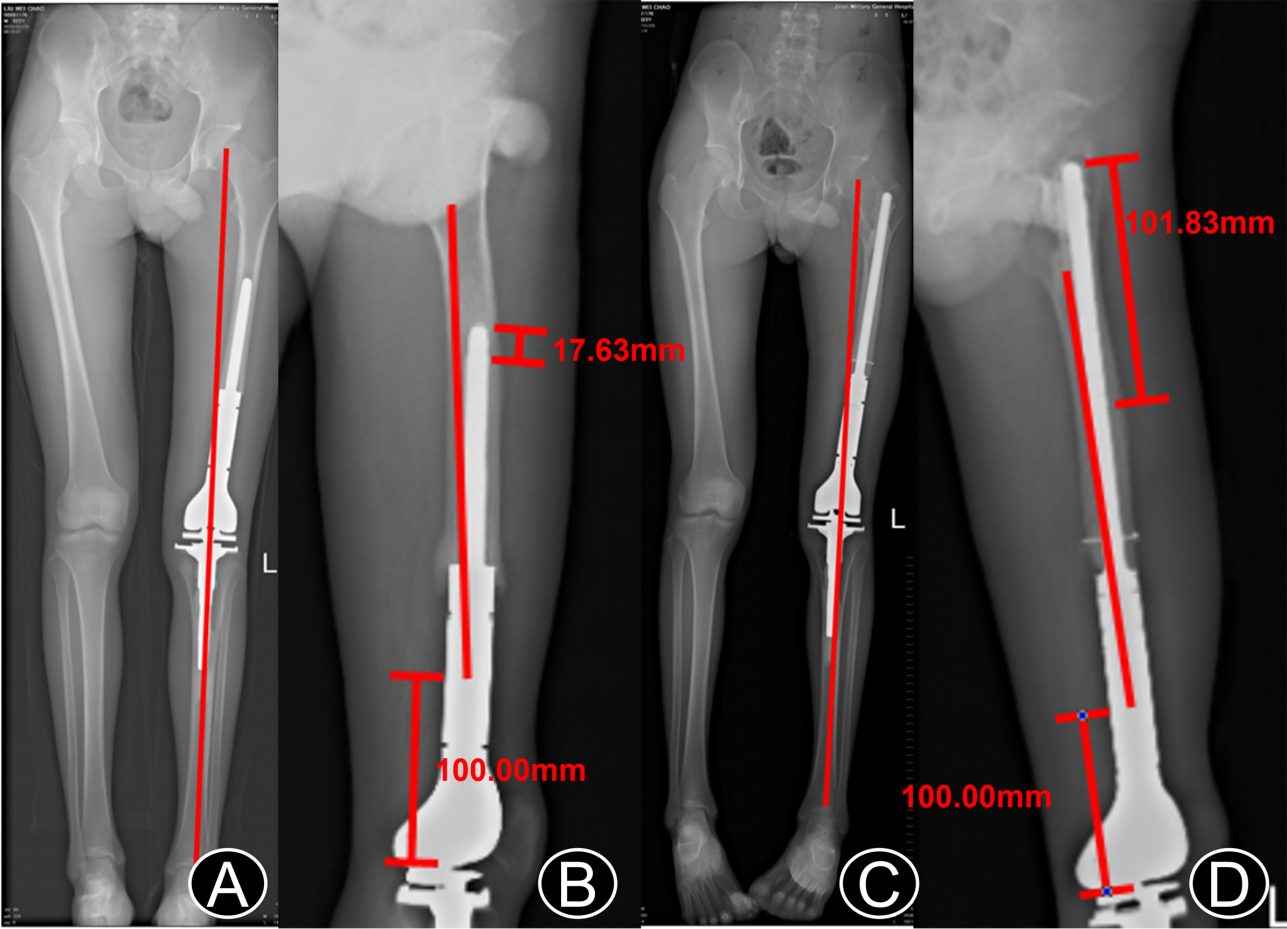
Figure A 22‐year‐old man with osteosarcoma of the distal left femur. (A) Lower limb strength line was found to be normal 1 month after the initial replacement. (B) Maximum distance between the apex of the stem and the anterior arch of the femur was 1.76 cm. (C) Imaging data after revision. A good force line of the lower limb was maintained. (D) A larger length of the medullary stem was selected so that the apex of the medullary stem was far away from the anterior femoral arch

Liu, a 22‐year‐old man, received tumor resection of the left distal femur and tumor‐type knee prosthesis replacement in our department on February 9, 2018, due to distal femur osteosarcoma. A postoperative review showed an aligned lower limb, but the vertex position was 1.763 cm away from the maximum arc of the anterior femoral arch (Figure 3A,B). Aseptic loosening occurred 9 months after the surgery. Revision surgery of the tumor‐type prosthesis was performed on December 13, 2018, during which the prosthetic stem was loosened and replaced with a prosthetic stem with a larger diameter and a longer length (Figure 3C), and the standard lower extremity force lines were maintained (Figure 3D). The patient had no subjective symptoms or objective imaging evidence of prosthetic loosening at the last follow‐up.

## Discussion

### Risk Factors for Aseptic Loosening

The stem of the femoral medullary cavity is a key component to achieve a firm connection between the entire prosthesis and the host femur^11^. This study indicated that the smaller ratio of the medullary stem diameter to the femoral diameter led to the aseptic loosening of the prosthesis. This result was the same as the conclusion of studies by Bergin *et al*.^18^ and Farfalli *et al*.^19^. Measurement and statistical analysis of the imaging data of the two groups of cases in this study showed that the ratio of the medullary stem diameter to the femoral diameter of less than 30% was a risk factor for aseptic loosening of the prosthesis. A smaller diameter percentage not only led to lower mechanical strength of the medullary cavity stem but also meant that a thicker bone cement needed to be filled between the medullary cavity stem and the medullary cavity, making the bone cement not only poor in torsion resistance but also nondegradable. The debris generated by the wear of bone cement might activate T cells and macrophages around the prosthesis and osteoclasts and cause osteolysis^20^, ^21^. Therefore, using a large amount of bone cement to fill the gap between the medullary canal stem and the bone cortex is not recommended; instead, a medullary canal stem that fits the thickness of the femoral medullary cavity from the beginning to reduce the loosening rate should be chosen^12^, ^22^.

The lower limb alignment is the “gravitational line of the legs” and the correct reconstruction of the limb alignment in total knee arthroplasty is an important factor affecting the quality of the surgery and the recovery of the knee function after surgery^23^, ^24^. Vanlommel *et al*.^25^ suggested that an appropriate varus knee was beneficial to the function of the knee joint, while Ritter *et al*.^26^ opined that a neutral position or mild knee valgus allowed for a longer prosthesis life. The lower limb alignment is equally important in oncologic prosthesis replacement. Seven patients in this study experienced knee varus or knee valgus, four of which had aseptic loosening. The statistical analysis of the data showed that a poor lower limb alignment increased the probability of the aseptic loosening of the prosthesis, and the distance of the lower limb alignment from the center of the knee after surgery positively correlated with the rate of loosening. In the case of abnormal lower limb alignment, the stress of the medullary stem on the wall of femoral bone marrow exceeded the maximum deformation value of cancellous bone, resulting in the stress concentration of the prosthesis and cortical bone, deformation of the medullary wall, and loosening and subsidence of the prosthesis. Meanwhile, the tilt of the force line was thought to accelerate the wear of the end of the medullary stem of the prosthesis against the femoral cortex and increase the loosening rate of the prosthesis by applying lateral–torsional forces during the normal operation of the prosthesis mechanics.

### Selection of Apex Position of the Prosthetic Stem

In this study, the position of the apex of the medullary stem was measured and analyzed. The results showed that the position of the apex of the medullary stem affected the loosening of the prosthesis, which was consistent with the study by Unwin *et al*.^27^. This study suggested that the relative distance of less than 3 cm between the apex of the medullary stem and the maximum curvature of the anterior femoral arch was a risk factor for the aseptic loosening of the prosthesis. On the contrary, if the relative distance between the two points was greater than 3 cm, the probability of the aseptic loosening of the prosthesis was smaller. This was related to the anterior femoral arch. Studies on the stress of the femoral prosthesis complex showed that femoral stress in tumor knee prosthesis replacement was concentrated near the apex of the medullary stem of the prosthesis^27^. The femur itself had a forward physiological curvature, and its anterior arch carried its own forward curvature that led to greater directional variation and stress concentration in the trabecular bone. When the apex of the femoral medullary stem was closer to the anterior femoral arch, the stress‐obscuring effect of the medullary stem occurred upon contact with the stress‐concentrated anterior femoral arch, causing local pressure concentration and loosening. Therefore, the apex of the medullary stem should be positioned away from the maximum femoral arc during replacement surgery, thereby reducing stress concentration to decrease the rate of prosthesis loosening. Batta *et al*.^28^ suggested that longer resection of the tumor segment and a relatively short stem could lead to the aseptic loosening of the distal femur, which might be related to uneven force and loss of surrounding bone under stress occlusion due to a short stem in the femoral bone marrow. We suggested that after determining the length of the osteotomy according to the preoperative examination, a longer medullary stem prosthesis should be selected with the apex of the medullary stem crossing the anterior femoral arch, so as to achieve better initial stability and reduce the loosening rate of the prosthesis.

### Recommendations for the Reduction of the Loosening Rate

This study suggested that the following surgical procedures should be performed in the clinical application of tumor‐type knee prosthesis replacement for treating a distal femoral malignant bone tumor. (i) The femoral prosthesis medullary stem with a larger diameter was selected and the amount of bone cement was reduced depending on the femoral diameter. Surgeons should attempt to maximize the canal filling of stems to obtain a solid press‐fit^29^. (ii) The femoral prosthesis with appropriate length was selected according to the location of the tumor and anterior femoral arch before surgery, with the apex of the medullary stem as far as 3 cm from the anterior femoral arch. (iii) According to the design of tumor‐type prosthesis, the femur in standard cross‐section was truncated, the extramedullary positioning was precise when performing tibial plateau osteotomy, and the physiological position was reconstructed as much as possible when placing the prosthesis in the medullary cavity to ensure the reconstruction of standard lower limb alignment. Bilateral lower extremity X‐rays were taken periodically postoperatively to observe whether the force lines were tilted.

### Limitations

This was a single‐center clinical study with a small sample size, which made it impossible to conduct an effective inter‐group study, thus having an impact on data analysis. Large‐sample statistics or laboratory measurements can be used to verify the conclusion of this study.

## Conclusions

In the postoperative radiographic measurement after tumor‐type knee prosthesis replacement, the diameter of the femoral medullary stem of the prosthesis, the position of the femoral bone marrow stem apex, and the lower limb alignment are the risk factors leading to aseptic loosening. For reducing the incidence of aseptic loosening, it is recommended to use a medullary stem that fits the diameter of the femur, select a longer medullary stem with its apex over the anterior femoral arch more than 3 cm, and reconstruct the standard lower extremity force line.

## Conflict of Interest

The tumor‐type knee prosthesis was used as the treatment method in this study, but all authors declared that they did not receive any funding for this device. The authors also declare no conflicts of interest in the research and writing process.
